# How Long Does it Last? The Enduring Benefits of Neurodiversity Training and Diagnostic Disclosure on Hiring Outcomes for Adults with ASD

**DOI:** 10.1007/s10803-025-06751-w

**Published:** 2025-02-18

**Authors:** Cynthia P. May, Christopher E. Whelpley, Levi Moyer, Lillian M. Feingold

**Affiliations:** 1 College of Charleston, 66 George St, Charleston, USA; 2 Virginia Commonwealth University, Richmond, USA; 3 Furman University, Greenville, USA

**Keywords:** Autism Spectrum Disorder (ASD), Employment, Diagnostic disclosure, ASD knowledge, Neurodiversity training, Double empathy gap

## Abstract

Even when perceived as highly qualified, job candidates with autism spectrum disorder (ASD) are less likely to be hired after a job interview than their neurotypical (NT) counterparts. However, when NT individuals receive training about neurodiversity and are aware of an ASD diagnosis, preliminary evidence suggests hiring outcomes for candidates with ASD are significantly more positive, at least when training occurs immediately before evaluation. This study examined whether the benefits of neurodiversity training coupled with diagnostic disclosure extend to the general population and persist over time. Participants included undergraduate students and individuals from the general U.S. population recruited through Prolific. They completed neurodiversity training either two weeks or two months before reviewing taped interviews of job candidates with and without ASD. They rated candidates on several social dimensions (e.g., trustworthiness, likeability, awkwardness) and indicated how likely they were to hire each candidate. Although candidates with ASD were rated less favorably on some social characteristics (e.g., awkwardness, similarity) relative to NT candidates, they were rated similarly on other dimensions (e.g., trustworthiness), and at both delays were just as likely to be hired as NT candidates. These findings suggest that evaluators who engage in neurodiversity training and receive diagnostic information about ASD candidates are favorably inclined to hire ASD candidates, and this positive disposition towards ASD candidates persists for several months after neurodiversity training.

First impressions are powerful and consequential. People are generally quick to make wide-ranging judgments about others, including judgments about competence, emotional stability, honesty, and likeability, often based on minimal information like appearance, gait, eye contact, and even handshake strength (Ambady & Rosenthal, 1992; Naumann et al., 2009; Norman & Goldberg, 1966; Stewart et al., 2008; Thoresen et al., 2012; Willis & Todorov, 2006). Although these impressions may be inaccurate, they nonetheless are impactful and enduring (De Keersmaecker & Roets, 2017; Ross et al., 1975; Thoresen et al., 2012; Wingate & Bourdage, 2019). With respect to employment, for example, evidence suggests that initial assessments impact hiring decisions and may be associated with promotion decisions for up to 6 years (Barrick et al., 2010; Black & Vance, 2021; Macan & Dipboye, 1988; Stewart et al., 2008; Swider et al., 2016).

The finding that first impressions can have a persistent influence on outcomes is particularly consequential for individuals with autism spectrum disorder (ASD), a condition characterized by differences in social interactive style that can include atypical facial expressions, eye contact, vocal prosody, gestures, and personal space (Bodfish et al., 2000; Cunningham & Schreiberman, 2008; de Marchena & Eigsti, 2010; Doherty-Sneddon et al., 2013; Edey et al., 2016; Faso et al., 2015; Grossman et al., 2010; Kennedy & Adolphs, 2014). Neurotypical (NT) observers do not always understand these differences (e.g., Szechy et al., 2023) and evidence suggests that as a result NT individuals judge people with ASD as odd, awkward, undesirable, or even deceptive (Dickter & Burk, 2021; Grossman et al., 2019; Lim et al., 2022; Sasson et al., 2017; Sasson & Morrison, 2019; Whelpley & May, 2023). These negative impressions are associated with a reluctance to engage people with ASD (e.g., Cage et al., 2019; Sasson et al., 2017) and can create barriers in the workplace (Ezerins et al., 2024; Scott et al., 2017).

One such barrier is the job interview. Growing research indicates that candidates with ASD are perceived less favorably than neurotypical candidates in job interviews, particularly if evaluators have little knowledge about ASD. For example, in one study participants read vignettes about neurotypical job candidates and job candidates with an ASD diagnosis who demonstrated characteristics associated with ASD (e.g., unusual eye contact, repetitive movements). Even in this paradigm in which autistic traits were described and not directly observed, candidates with ASD were rated less favorably than neurotypical candidates on measures including hirability and emotional stability (McMahon, Henry, & Linthicum, 2021). Similarly, in another study, evaluators who watched mock job interviews of candidates with and without ASD rated the candidates with ASD less favorably than NT candidates on social traits like awkwardness, likeability, and enthusiasm. Moreover, those unfavorable social ratings weighed heavily on hiring decisions for ASD candidates. Specifically, even when candidates with ASD were perceived as highly qualified, they were significantly less likely to be hired than NT candidates (Whelpley & May, 2023). Other studies in which participants observed candidates with and without ASD further demonstrate that ASD candidates are perceived less favorably and that evaluators are less inclined to hire them relative to NT candidates, particularly when evaluators are naïve to an ASD diagnosis and/or have little knowledge about ASD (Flower et al., 2021; Norris et al., 2024).

These research findings suggest that adults with ASD may have difficulty obtaining employment, and unfortunately the extant employment data bear this out. The majority of individuals with ASD are unemployed or underemployed, with disproportionately poor employment outcomes even when considering only individuals with disabilities (Howlin, 2013; Neary et al., 2015; Roux et al., 2013, 2015; Taylor et al., 2015). Low employment rates for individuals with ASD exist even after controlling for intelligence and educational attainment (Howlin & Moss, 2012). Indeed, when considering only individuals with ASD who do not have an intellectual disability (ID), estimates are that less than 60% of those individuals are employed full-time or enrolled in postsecondary education (Neary et al., 2015; Roux et al., 2015; Taylor et al., 2015), even though many autistic people have strong ambitions to work (Baldwin et al., 2014). Given that most employers still use job interviews as the principal gateway for employment, and that highly qualified candidates with ASD are disadvantaged in traditional job interviews, an essential step in solving the employment equation for adults with ASD is the development of ways to overcome the inequities inherent in the interview process.

A number of alternatives have been explored to mitigate the influences of negative first impressions of people with ASD in the interview process. One approach emphasizes training prospective employees with ASD to alter some of the traits or behaviors commonly associated with autism. This approach focuses on identifying specific aspects of the social interaction that drive the negative impressions of adults with ASD (e.g., eye contact, body posture, responses to interview questions) and addressing those behaviors through training for individuals with ASD. Preliminary evidence suggests that training programs for adults with ASD may indeed be successful in improving nonverbal communication skills, understanding others’ point of view, generating appropriate responses to interview questions, and self-confidence (e.g., Horn et al., 2023; Kumazaki et al., 2019a, b). However, this strategy for leveling the playing field for prospective employees with ASD may not provide a comprehensive solution for several reasons. First, this approach is unlikely to prove sufficient in fully addressing the challenges faced by prospective employees with ASD, as evidence suggests that the negative evaluations of individuals with ASD are not driven by a single difference or a collection of specific differences, but rather by the individual’s overall presentation (e.g., Lim et al., 2022; Sasson et al., 2017). Second, this approach may also require individuals with ASD to mask their autistic traits, which could make a stressful interview even more difficult (e.g., Bradley et al., 2021). Third, recent research acknowledges the fact that social interactions are bidirectional and thus the challenges faced by individuals with ASD stem not only from their atypical social style but also from biases held by NT individuals (Norris et al., 2024). Known as the double empathy gap, this barrier can result in the breakdown of communications between autistic and neurotypical individuals due to misinterpretation of social signals between parties (Milton, 2012). For example, differences in expectations, social style, and experiences between ASD and NT individuals could result in lower interview performance for autistic individuals being judged by NT interviewers (e.g., Milton, 2012; Milton et al., 2018; Scheerer et al., 2022). Bridging this gap may be essential for long-term improvement in employment outcomes for adults with ASD.

Evidence suggests that one approach for addressing the double empathy gap is to encourage candidates with ASD to disclose their diagnosis and to educate employers about ASD. Indeed, ratings of people with ASD are often more favorable when evaluators are aware of their diagnosis (Butler & Gillis, 2011; Flower et al., 2021; Lim et al., 2022; Maras et al., 2019; Matthews, Ly, & Goldberg, 2015; Norris et al., 2024; Sasson & Morrison, 2019). Providing a diagnostic label may help observers understand and contextualize atypical behavior (e.g., differences in eye contact; Brosnan & Mills, 2016; Sasson & Morrison, 2019). In one study of 254 adults with ASD, those who disclosed their diagnosis to their employers were more than three times as likely to be employed than those who did not disclose (Ohl et al., 2017). That said, many employees with ASD are reluctant to disclose their diagnosis to everyone in the workplace over concerns about how it will affect co-workers’ attitudes towards them. In another study of 238 autistic adults, only a third of those who disclosed to their supervisors rated the impact positively (Romualdez et al., 2021). Indeed, research suggests that disclosure can backfire if evaluators do not have strong knowledge about ASD (Morrison et al., 2019; Whelpley et al., 2021), and that the stigma associated with autism is often heightened among individuals with limited knowledge about the disorder (Ling et al., 2010; Mahoney, 2007; Someki et al., 2018). Thus, disclosure in the absence of additional information about ASD may not be an optimal solution.

Both employees with ASD and managers have noted that the experience for those with ASD would improve significantly if employers received training that offered an enhanced understanding of autism and its complexities (Whelpley et al., 2021). In line with this suggestion, impressions of people with ASD are significantly more positive when raters have greater understanding about ASD, either through education or interpersonal contact (e.g., Dachez & Ndobo, 2018; Dickter & Burk, 2021; Gardiner & Iarocci, 2014; Gillespie-Lynch et al., 2022; Lu et al., 2022; McMahon et al., 2021; Morrison et al., 2019; Obeid et al., 2015; Sasson & Morrison, 2019; Someki et al., 2018). Growing evidence suggests that combining these two approaches, that is pairing diagnostic disclosure with accurate ASD knowledge, may be necessary (e.g., Brosnan & Gavin, 2021; McMahon et al., 2021; Morrison et al., 2019; Whelpley et al., 2021).

There is suggestive evidence that hiring outcomes for individuals with ASD are better with this dual approach of pairing increased ASD knowledge with diagnostic disclosure. For example, McMahon et al. (2021) asked participants to assess the employability of vignette characters with and without ASD and found that participants with more knowledge about ASD had more positive perceptions of candidates with ASD, particularly when an ASD diagnosis was explicit. In another study, providing an ASD diagnosis improved raters’ perceptions of interviewees, and providing additional information about ASD reduced the hiring disadvantage for candidates with ASD (Flower et al., 2021). That said, Norris and colleagues found that while diagnostic disclosure improved perceptions of job candidates with ASD, providing additional information about autism did not further improve ratings (Norris et al., 2024). One limitation of these studies is the fact that the additional information provided about autism was somewhat limited. McMahon et al. simply measured evaluators’ knowledge about ASD rather than providing specific information about autism, and thus even individuals with “high” autism knowledge in their study may have differed in their understanding of specific traits or behaviors associated with autism or the complexity of the spectrum. Participants in the Flower et al. study who received “detailed” information about autism read a paragraph of less than 100 words, while those in the Norris et al. study read a one-page document (less than 400 words) that included differences in verbal and non-verbal behaviors and communication style. Neither study assessed knowledge about ASD after the presentation of the additional information to confirm comprehension and retention.

A recent study by May and colleagues (May et al., 2025) assessed the impact of a more intensive approach to increasing raters’ knowledge about autism and pairing it with diagnostic disclosure. Expanding on their earlier work (Whelpley & May, 2023), May et al. had participants evaluate job interview videos that included candidates with and without ASD. The videos were identical to those used by Whelpley and May (2023), who found that candidates with ASD were rated poorly and were less likely to be hired relative to NT candidates. Prior to reviewing these videos, participants in the May et al. study completed a 30-minute online neurodiversity training. The training, which was based on the work of by Gillespie-Lynch and colleagues (Gillespie-Lynch et al., 2022; Waisman et al., 2023), featured several video clips of adults with ASD sharing their experiences and perspectives as individuals on the spectrum. The training reviewed characteristics commonly associated with autism, debunked several myths about autism, and offered suggestions for supporting individuals with ASD in the workplace. After viewing the online training and passing two comprehension checks, participants in the May et al. study rated the interview videos. Before each video, participants received diagnostic information about each candidate. Thus, participants knew in advance whether each job candidate had ASD or was NT. The findings from this training study diverged from those of Whelpley and May (2023) in a few important ways. First, although the candidates with ASD continued to be rated less favorably on some social dimensions (e.g., awkwardness, attractiveness, likeability) relative to NT candidates, they were not rated less favorably on other social dimensions (e.g., trustworthiness, confidence, enthusiasm, straightforwardness). Most significantly, there were no differences in participants’ likelihood of hiring ASD candidates versus NT candidates.

Together, these findings offer hope that increased knowledge about ASD, coupled with diagnostic disclosure, may provide a promising approach to correcting bias against individuals with ASD in the job interview process. However, there are some limits to the extant research. In the May et al. (2025) study, the participants were all undergraduate students enrolled in postsecondary education. Because these students were engaged in ongoing higher education, they may have been more open to new ideas or perspectives than the general population, and so the neurodiversity training may have been particularly impactful for them. It is important, therefore, to include a broader sample of participants from the general population to ascertain whether the training has widespread benefits with a more diverse audience. In addition, in all the studies that provided enhanced information about autism, the information was presented immediately before participants evaluated the NT or ASD candidates (Flower et al., 2021; May et al., 2025; Norris et al., 2024). In everyday work settings, diversity training typically takes place weeks or even months, not minutes, before employers engage in the hiring process. It would be useful to explore whether the benefits of additional ASD knowledge persist over longer periods of time.

The present investigation was designed to address these issues. First, we recruited participants from two different samples. We again included undergraduate students from a southeastern university so that we could determine whether the findings from May et al. (2025) replicate with a longer delay. We also recruited participants using the Prolific online platform. Here, we recruited adults between the ages of 18 and 55 years from across the United States. Doing so allowed us to test a sample that was more diverse in terms of educational background, age, gender, geographic residence, and race. Second, we expanded the timeframe of the study, inserting a two-week delay for some participants and a two-month delay for others. Pilot testing using the Prolific platform suggested significant participant attrition beyond a two-week window. Thus, to optimize study completion and integrity of the data, we used a two-week delay for the Prolific participants. To determine whether the benefits of neurodiversity training persist for longer durations, we used a two-month delay for the undergraduate participants. All participants completed the same neurodiversity training used by May et al. (2025), and then watched and evaluated job candidates with and without ASD after a delay. Prior to reviewing each candidate, raters were informed whether the candidate did (or did not) have a diagnosis of ASD. To preview the findings, our results were generally consistent with those from May et al. in demonstrating less favorable ratings for ASD relative to NT candidates on some (but not all) social measures. Most significantly, there were no differences in ratings of hirability across the candidate groups at either delay.

## Method

### Participants

Three hundred and one participants completed the study, and all participation was voluntary. Ninety-six participants were residents of the United States (ages 19–55 yrs.) who were recruited through the Prolific online platform and received modest compensation for their participation. The majority of the Prolific sample (53%) identified as female, 46% as male, and 1% preferred not to report gender identity. All Prolific participants had earned at least a high school diploma or its equivalent, and the majority (76%) earned either an additional technical degree (8%) or college diploma (68%). The majority of our Prolific sample (64%) self-reported as Caucasian, 21% identified as Black, 9% identified as Asian, and the rest preferred not to identify. All participants recruited through Prolific first completed the online neurodiversity training and then waited two weeks before completing the interview evaluation portion of the study.

Two hundred and five participants were undergraduate students at a southeastern university in the United States (ages 18–25 yrs.) who completed the study as one way to fulfill a course requirement. The majority (79%) of the undergraduate sample identified as female, 19% identified as male, and 2% identified as non-binary. Data on race/ethnicity were not recorded.

### Materials

#### Neurodiversity Training

Our neurodiversity training videos were adapted from training developed by Gillespie-Lynch and colleagues (Gillespie-Lynch et al., 2022; Waisman et al., 2023). Their original training was created in a collaborative process that included autistic individuals and was designed to educate college professors about ASD and address ways to decrease stigma within the university setting. We adapted the training to educate individuals about ASD, increase awareness and understanding of some of the common traits and experiences of people on the spectrum, and reflect the value of neurodiversity in the workplace. To this end, we adapted their materials to create two training videos (each approximately 14 min).

The first training video provided an overview of what the term neurodiversity means and listed the criteria for a DSM-5 diagnosis for ASD. The segment included a video of two college students with ASD who talked about their first-hand experiences with ASD and described various behaviors and traits with which they self-identified, including resistance to change, differences in communication, and stimming behaviors. The video finished with an overview of characteristics of ASD, including social and communication differences, approaches to learning and sensory differences.

The second training video included insights from a professor who shared her experiences as a person on the spectrum. The professor discussed the myths about people with ASD from a first-hand point of view, debunked those myths, and then described how to better support someone with ASD. This video also included information correcting the common misconceptions about ASD and recommendations for allies of the neurodiverse community.

#### Job Interview Videos

For the video-rating task, we used the same videos employed by Whelpley and May (2023). Thirty college students (15 ASD/15 NT) were videotaped performing a mock job interview. All interviewees were degree-seeking undergraduates (ages 18–25 yrs.) who matriculated through the regular admissions process at one of two universities on the East Coast of the United States. The students with ASD were recruited from the Disability Services Office (DSO) at their respective universities and were offered modest compensation. All interviewees with ASD had a confirmed diagnosis of autism on record with the DSO at their institution. Consistent with similar studies examining impressions of adults with ASD (e.g., Sasson et al., 2017; Sasson & Morrison, 2019), none of the interviewees with ASD had dual diagnosis of intellectual disability. The ASD cohort included nine individuals who identified as female and six as male. Eleven were Caucasian, two Latinex, and two African American. The NT interviewees were recruited from introductory psychology courses and were offered the same compensation provided to ASD interviewees. None of the NT candidates had a diagnosis of ASD. Eleven of the NT candidates identified as female and four as male. The NT candidates included 11 Caucasian interviewees, three Latinex, and one African American. All participation was voluntary, and interviewees were informed that they could withdraw from the process at any time.

For the interview task, each interviewee was asked to imagine that they were being interviewed for their dream job. They were told to prepare a 5-minute speech explaining why they would be qualified for the job and were given 5 min to prepare their answer. If an interviewee stopped talking before the full 5 min elapsed, the experimenter requested, “please continue.” Interviewees were aware that their responses would be videotaped and used for research purposes. Video duration ranged from 175 s to 300 s, with an average time of 268 s. The average interview time was 271 s for NT candidates and 265 s for ASD candidates. The 30 videos were divided into three groups of 10, with five videos from NT candidates and five videos from candidates with ASD in each group. Testing was designed so that each group of 10 videos was used roughly an equal number of times across participants.

### Procedure

#### Neurodiversity Training

All participants completed the study online using the Qualtrics platform. For Phase 1 (neurodiversity training), participants gave informed consent and then watched the first training video. The program ensured that the training video played in its entirety, at regular speed. A five-item quiz followed. If participants did not earn at least 80% on the quiz, they were directed back to the training video. Once participants successfully completed the first video and quiz, they watched the second video and completed an additional quiz with the same parameters.

When participants watched both videos and passed both quizzes, they received an email confirming completion of the training and notifying them of the start date for the second phase of the study. Prolific participants were informed that they would receive a link to Phase 2 after two weeks, while undergraduate students were informed they would receive the link after two months.

#### Video Evaluation

After the designated delay period, participants received a Qualtrics link via email that they could use to watch and evaluate the mock job interviews. Participants were given a two-week window in which to complete Phase 2. Thus, Prolific participants completed Phase 2 between two and four weeks after training, while undergraduate participants completed Phase 2 between nine and eleven weeks after training. Reminder emails were sent periodically during this period to increase participation rates. For each participant, 10 videos (half with ASD candidates) were presented in random order. Before each candidate video, participants were informed as to whether the candidate did (or did not) have an ASD diagnosis.

The program was set so that participants had to watch each video in its entirety, at regular speed, before evaluating a candidate. In line with other research (Sasson et al., 2017; Whelpley & May, 2023), interviewees were evaluated on ten measures: likability, trustworthiness, straightforwardness, job qualifications, attractiveness, awkwardness, confidence, enthusiasm, captivation, and similarity to oneself. For each measure, participants read a statement such as, “This candidate is TRUSTWORTHY” and rated their agreement with the statement on a 7-point Likert scale, with 1 = strongly agree and 7 = strongly disagree. In addition, one foil question was included for each candidate to ensure that participants read each question carefully. The foil question read, “Please respond “strongly agree” so that we know you are paying attention.” Items of this sort have been noted as a straightforward method of identifying respondents who are paying attention and complying with instructions (DeSimone & Harms, 2018). All items appeared in random order across participants to avoid any ordering bias.

Two additional questions were asked after those ten social measures. First, raters responded to the statement, “The overall performance of this individual was good” by rating the candidate on a Likert scale where 1 = strongly agree and 7 = strongly disagree. Second, in line with Cable and Judge (1997), raters responded to the statement, “I would hire this individual for the job they described” using a Likert scale where 1 = strongly agree and 7 = strongly disagree. This process was repeated until all ten candidates were evaluated.

## Results

Prior to performing any analyses, we examined our data for outliers, including participants who gave the same score for all interviewees and measures (e.g., repeatedly pressing the “7” key). We also identified individuals who failed to respond appropriately to a foil question during the interview rating task. Data from 16 participants (one Prolific participant and 15 undergraduate students) were excluded from analyses as a result. Our findings are thus based on data from 285 raters (95 Prolific participants and 190 undergraduate students). Ratings were reverse scored so that for all measures (excluding awkwardness), higher scores reflected more favorable ratings (e.g., more attractive, more likely to hire). For awkwardness, lower scores reflect less awkwardness.

We conducted a 2 (Interview Group: NT vs. ASD) x 2 (Delay: 2-week vs. 2-month) X 11 (Measure) mixed measures ANOVA. Results showed a significant main effect of Interview Group, *F* (1, 283) = 6.6, *p* =.01, η^2^ = 0.02, with higher ratings overall for the NT candidates (*M* = 3.7) relative to the ASD candidates (*M* = 3.5). There was also a significant main effect of Measure, *F* (10, 2830) = 179.4, *p* <.001, η^2^ = 0.39, indicating that scores varied significantly across our different measures. There was no main effect of Delay, *F* < 1.

The main effects were qualified by several interactions, including reliable interactions between Interview Group and Measure, *F* (10, 2830) = 53.0, *p* <.001, η^2^ = 0.16, Interview Group and Delay, *F* (1, 283) = 4.0, *p* =.046, η^2^ = 0.01, and Delay and Measure, *F* (10, 2830) = 2.8, *p* <.001, η^2^ = 0.01. Finally, there was a 3-way Interview Group X Delay X Measure interaction, *F* (10, 2830) = 3.4, *p* <.001, η^2^ = 0.01.

**Fig. 1:**
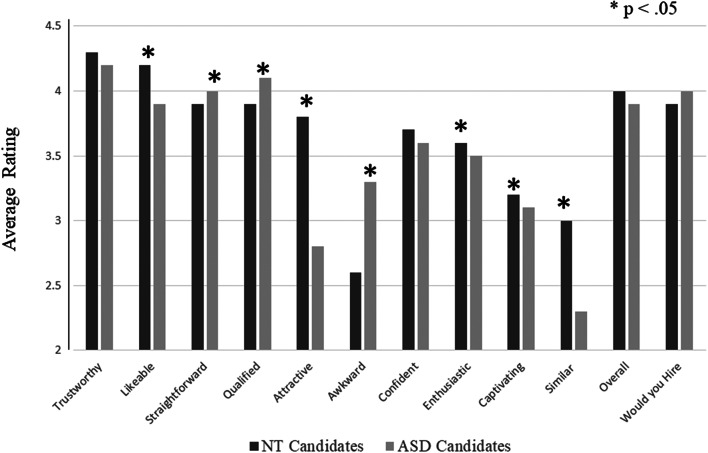
Average ratings for NT candidates and candidates with ASD across Delays

Further analyses were conducted to discern whether the ratings for NT candidates differed from ASD candidates for each measure, first collapsed across delays and then for each delay separately. As can be seen in Fig. 1, when ratings were collapsed across delays, NT interviewees were rated more favorably than ASD interviewees on a number of social dimensions, including likeability, *t*(284) = 5.1, *p* <.001, attractiveness, *t*(284) = 14.8, *p* <.001 awkwardness, *t*(284) = 10.4, *p* <.001, enthusiasm, *t*(284) = 2.2, *p* =.03, captivating, *t*(284) = 3.0, *p* <.01, and similarity, *t*(284) = 9.6, *p* <.001. On two social dimensions, however, there were no significant differences between candidates with ASD and without ASD. These measures were trustworthiness, *t*(284) = 1.0, *p* =.3, and confidence, *t* < 1. Furthermore, candidates with ASD were perceived more favorably than NT candidates on measures of straightforwardness, *t*(284) = 2.1, *p* =.04, and qualifications, *t*(284) = 2.8, *p* <.01. Most central to our purposes, there were no differences between NT candidates and those with ASD on overall ratings, *t* < 1, or on the likelihood of being hired, *t* < 1.

**Fig. 2:**
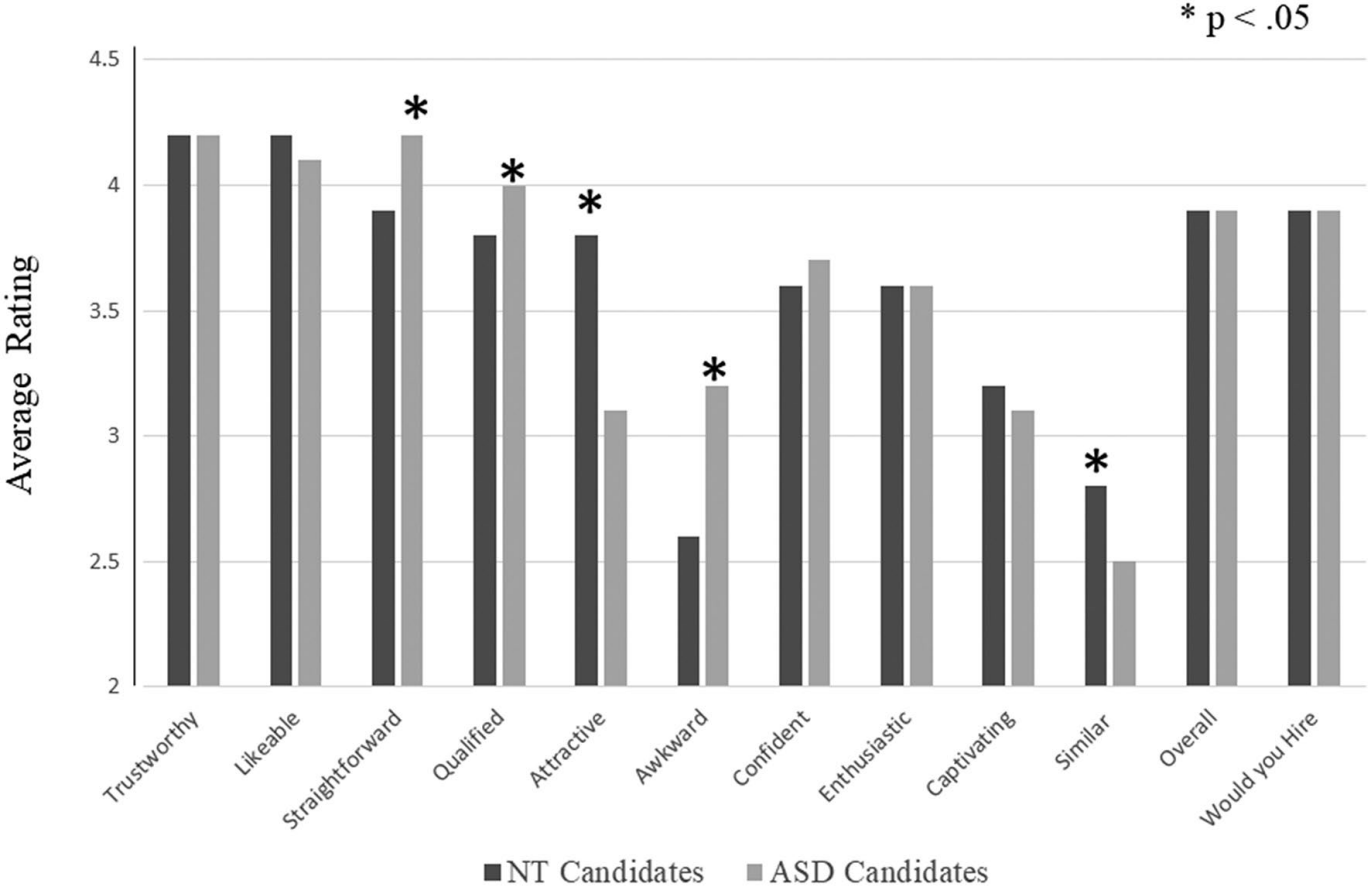
Average ratings for NT candidates and candidates with ASD at the 2-week Delay

Next we explored whether the ratings for the NT and ASD group differed across measures for the Prolific participants tested at the 2-week delay. As can be seen in Fig. 2, the pattern of findings for the Prolific group at the 2-week delay was similar, but not quite identical, to the collapsed findings. Considering only the Prolific participants tested at the 2-week delay, a 2 (Interview Group: NT vs. ASD) X 11 (Measure) repeated measures ANOVA indicated no significant effect of Interview Group, F < 1, a significant effect of Measure, F (10, 940) = 44.8, *p* <.0001, η^2^ = 0.32, and a significant Interview Group X Measure interaction, F (10, 940) = 14.6, *p* <.0001, η^2^ = 0.13. Paired samples t-tests indicated that NT interviewees were rated more favorably than ASD interviewees on three social dimensions, including attractiveness, *t*(94) = 6.8, *p* <.001 awkwardness, *t*(94) = 4.7, *p* <.001, and similarity, *t*(94) = 2.6, *p* <.01. On several other social dimensions, however, there were no significant differences between candidates with and without ASD. These measures were trustworthiness, *t* < 1, likeability, *t*(94) = 1.1, *p* =.29, confidence, *t* < 1, enthusiasm, *t* < 1, and captivating, *t*(94) = 1.1, *p* =.26. As with the data collapsed across delays, at the 2-week delay candidates with ASD were perceived more favorably than NT candidates on measures of straightforwardness, *t*(94) = 2.7, *p* <.01, and qualifications, *t*(94) = 2.2, *p* =.03. Furthermore, at the 2-week delay with the Prolific sample, there were no differences between NT candidates and those with ASD on overall ratings, *t* < 1, or on the likelihood of being hired, *t* < 1.

**Fig. 3:**
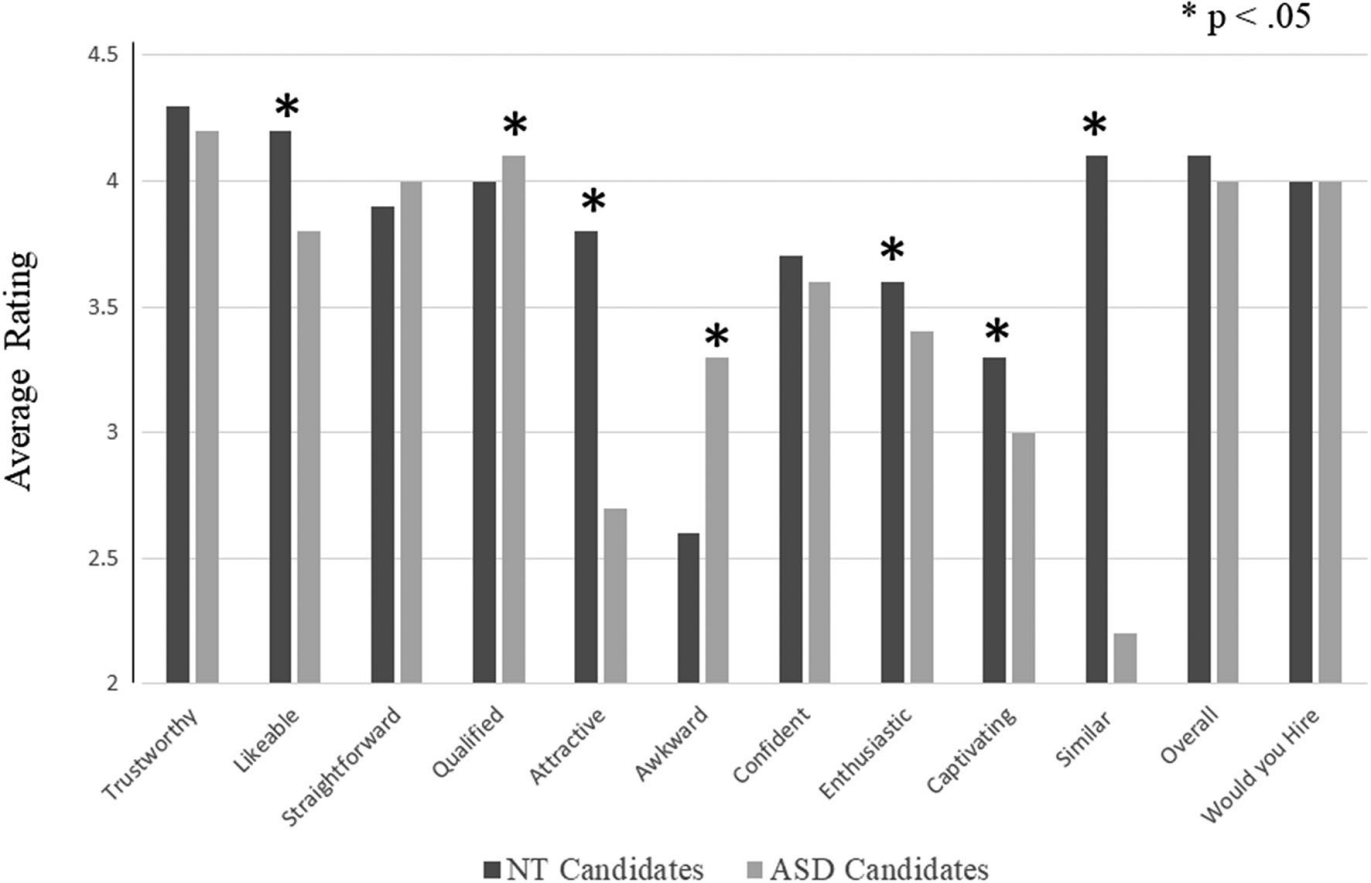
Average ratings for NT candidates and candidates with ASD at the 2-month Delay

Finally, we examined the ratings for ASD versus NT candidates for undergraduates tested at the 2-month delay. A 2 (Interview Group: NT vs. ASD) X 11 (Measure) repeated measures ANOVA indicated a significant effect of Interview Group, *F* (1, 189) = 6.5, *p* =.012, < 1, η^2^ = 0.03, a significant effect of Measure, *F* (10, 1890) = 108.4, *p* <.0001, η^2^ = 0.36, and a significant Interview Group X Measure interaction, *F* (10, 1890) = 49.9, *p* <.0001, η^2^ = 0.21. As can be seen in Fig. 3, NT interviewees were rated more favorably than ASD interviewees on a number of social dimensions, including likeability, *t*(189) = 5.5, *p* <.001, attractiveness, *t*(189) = 13.5, *p* <.001 awkwardness, *t*(189) = 9.4, *p* <.001, enthusiasm, *t*(189) = 2.4, *p* =.03, captivating, *t*(189) = 2.9, *p* <.01, and similarity, *t*(189) = 10.2, *p* <.001. There were no significant differences between ASD and NT candidates on measures of trustworthiness, *t*(189) = 1.2, *p* =.22, confidence, *t*(189) = 1.5, *p* =.13, or straightforwardness, t < 1. Candidates with ASD were perceived as marginally more qualified than NT candidates, *t*(189) = 1.9, *p* =.06. Finally, for undergraduate raters at the 2-month delay, there were no differences between NT candidates and those with ASD on overall ratings, *t*(189) = 1.4, *p* =.18, or on the likelihood of being hired, *t* < 1.

Using code provided by Tonidandel and LeBreton (2015), we performed a relative weights analysis to understand the importance of the different ratings with respect to predicting the likelihood of hiring. We note that we can compare relative weights within, but not across, models in terms of statistical significance and, consequently, cross model comparisons are directional. Table 1 shows the relative weights for the different ratings for ASD and NT candidates at both the two-week delay and the two-month delay. Although the relative weights for some of the social dimensions (e.g., trustworthiness, captivating) varied somewhat across candidate groups and delays, we note that candidates’ perceived qualification was similarly predictive of hiring scores for both groups. This was true at both the two-week delay and the two-month delay.

**Table 1:** Relative weights analysis of predictors of likelihood hiring decision

	2-week delay				2-month delay			
	ASD group		NT group		ASD group		NT group	
	Raw weight	Relative weight	Raw weight	Relative weight	Raw weight	Relative weight	Raw weight	Relative weight
Trust	0.09	12.56	0.09	13.12	0.13	18.66	0.06	9.58
Liking	0.09	12.85	0.06	9.16	0.09	13.27	0.08	11.99
Straightforward	0.1	14.76	0.07	9.77	0.08	11.5	0.07	10.93
Qualified	0.14	20.46	0.15	21.19	0.18	27.34	0.13	19.76
Attractiveness	0.04	5.43	0.04	5.65	0.02	3.01	0.03	3.81
Awkwardness	0.03	4.12	0.02	2.64	0.02	3.65	0.06	8.83
Confident	0.05	7.84	0.03	4.52	0.07	10.51	0.03	4.22
Enthusiastic	0.04	5.95	0.1	13.87	0.03	4.85	0.09	14
Captivating	0.11	16.03	0.14	20.08	0.05	7.21	0.11	16.88
Total R-Squared	0.69		0.69		0.67		0.66	

## Discussion

Job candidates with ASD are often perceived as socially awkward and less likeable than NT candidates, and these social deficits can adversely affect hiring outcomes (e.g., Cage et al., 2019; DeBrabander et al., 2019; Morrison et al., 2019; Norris et al., 2024; Sasson et al., 2017; Sasson & Morrison, 2019; Scott et al., 2017; Whelpley & May, 2023; Wingate & Bourdage, 2019). Recent work suggests that biases against candidates with ASD may be reduced when evaluators receive additional knowledge about ASD immediately prior to reviewing job candidates and are aware of a candidate’s ASD diagnosis (Flower et al., 2021; May et al., 2025; McMahon et al., 2021). The present study examined whether the benefits of this interventional strategy persist over time and generalize to a diverse sample of evaluators. We recruited evaluators from the general U. S. population using the online Prolific platform as well as undergraduate students at an American university. Prolific participants completed neurodiversity training two weeks prior to reviewing interview videos of job candidates with and without ASD, while the undergraduate participants completed the neurodiversity training two months before evaluating job candidates. During the evaluation process, all raters were informed whether each candidate had (or did not have) an ASD diagnosis before reviewing each video. Evaluators rated candidates on a number of social dimensions (e.g., likeability, trustworthiness, awkwardness) and indicated how likely they were to hire each candidate.

Both the Prolific and undergraduate participants rated candidates with ASD less favorably than NT candidates on some of the social measures, though the pattern of findings differed somewhat across the two rater groups. Consider first the data from Prolific participants, who rated job candidates two weeks after completing neurodiversity training. Prolific participants rated candidates with ASD as more awkward, less similar to themselves, and less attractive relative to NT candidates. However, candidates with ASD were rated as favorably as NT candidates on measures of trustworthiness, likeability, confidence, enthusiasm, and captivating. Furthermore, candidates with ASD were rated more favorably than NT candidates on measures of straightforwardness and qualifications. Finally and most significantly, hiring ratings did not differ across ASD and NT candidates, and candidates’ perceived qualifications were similarly predictive of hiring scores for both ASD and NT interview groups.

The findings from the Prolific participants tested at the two-week delay differ from those of Whelpley and May (2023), whose evaluators received no additional information about autism and were unaware that any of the candidates they reviewed had ASD. Whelpley and May found that ASD candidates were rated less favorably than NT candidates on all social measures except for “qualified,” and were less likely to be hired than NT candidates. The more positive social ratings observed for Prolific participants at the two-week delay in the present study, when evaluators received neurodiversity training coupled with diagnostic disclosure, echo recent findings that demonstrate that knowledge about ASD and/or disclosure of an ASD diagnosis can soften some of the negative social impressions about people with ASD (Gillespie-Lynch et al., 2015, 2022; Lu et al., 2022; McMahon et al., 2021; Obeid et al., 2015; Sasson & Morrison, 2019; Someki et al., 2018; Waisman et al., 2023). The fact that candidates with ASD were rated as equally likely to be hired relative to NT candidates replicates the findings of May et al. (2025) in showing that neurodiversity training coupled with diagnostic disclosure can level the playing field for candidates with ASD, even when those candidates are perceived as more awkward, less attractive, and less similar to the employers. Furthermore, it extends these findings by demonstrating that the benefits of neurodiversity training persist for several weeks and generalize to raters selected from the general population.

The data from the undergraduate participants tested at the two-month delay mirror this pattern, with some exceptions. When undergraduate evaluators reviewed job candidates two months after completing neurodiversity training, they rated candidates with ASD less favorably than NT candidates not only with respect to awkwardness, attractiveness, and similarity, but also on measures of likeability, captivating, and extraversion. Candidates with ASD were rated as equally trustworthy, confident, and straightforward as NT candidates, and were rated as marginally more qualified than NT candidates. Most significantly, even when training occurred two months before candidate evaluation, undergraduate raters were equally likely to hire candidates with ASD and NT candidates. These findings provide evidence for an enduring benefit of the combination of neurodiversity training and diagnostic disclosure.

The differences in the social ratings across the delays – with ASD candidates rated less favorably than NT candidates on a few more measures with a longer delay between training and evaluation sessions – could reflect a waning of the impact of the neurodiversity training. However, we believe it is more likely to reflect differences in the sample of raters. Recall that the raters at the two-week delay were drawn from the general population, while those at the two-month delay were undergraduate students. May et al. (2025) also used undergraduate students as raters, and although there was no delay between the neurodiversity training and the evaluation task in that study, candidates with ASD in their study were also rated less favorably on many social dimensions relative to NT candidates. It is possible that undergraduate students have higher social expectations and thus were more critical social raters relative to our Prolific sample, which was more diverse than the undergraduate sample in several ways (e.g., age, gender, and educational background). Unfortunately, our design does not permit a direct analysis of this question. However, further evidence that the differences in social ratings across our two delays were driven by differences in the raters rather than a waning of the training is the finding that at the longer delay, ASD candidates were still equally likely to be hired as NT candidates. Thus, the benefits of the neurodiversity training, at least with respect to hiring outcomes, were robust after two months.

### Limitations

There are some limitations of the present work that should be considered as employers strive for more equitable approaches to hiring neurodiverse adults. First, we did not include a pre-test in which participants viewed and evaluated job candidates before receiving neurodiversity training or a control condition in which participants received no training, and thus we were not able to document an improvement in ratings for ASD candidates as a direct result of the training or diagnostic disclosure. Although robust evidence documents the fact that without knowledge about ASD or awareness of an ASD diagnosis, neurotypical raters tend to perceive individuals with ASD unfavorably on social and hiring dimensions (Dickter & Burk, 2021; Flower et al., 2021; Grossman et al., 2019; Lim et al., 2022; Norris et al., 2024; Sasson et al., 2017; Sasson & Morrison, 2019; Whelpley & May, 2023), the absence of a control condition here prevents a direct assessment of the extent of the improvement in outcomes as a result of our intervention.

Additionally, the mock job interviews used in this study were somewhat artificial in the sense that candidates were asked to discuss their qualifications for their dream job, and jobs varied by candidate. In addition, the interviews were minimally interactive relative to traditional face-to-face interviews, which often require candidates to discuss not only strengths and qualifications but also weaknesses and challenges. Because candidates with ASD differ from NT candidates not only in their nonverbal behaviors and social style but also in their responses to questions (e.g., staying on topic, being overly direct, providing a specific level of detail), it may be beneficial for candidates with ASD to receive support or coaching for crafting appropriate responses to common interview questions (Horn et al., 2023).

The neurodiversity training used in this study was fairly intensive in that it required roughly 30 min to complete and included two knowledge tests, both of which required a score of 80%. We adapted our training from that used by Gillespie-Lynch and colleagues (Gillespie-Lynch et al., 2022; Waisman et al., 2023) who, using similar training with educators from 53 institutions across five countries, demonstrated benefits of the training that persisted for at least a month (Waisman et al., 2023). The training featured the voices of autistic adults sharing their experiences on the spectrum and afforded our participants the opportunity not only to learn basic facts about autism spectrum disorder but also to observe different individuals with ASD, who varied in their social style and in their autistic traits. It is not clear whether the enduring benefits observed here derived from the extensive information presented about ASD, the ability to hear and see different adults on the spectrum, or both. Regardless, our training is certainly more extensive and time-demanding than the material used in some studies to increase knowledge about ASD (e.g., Flower et al., 2021; Norris et al., 2024), and further research should explore whether this additional richness of detail and content is essential for lasting benefits.

Our data suggest that the impact of neurodiversity training persists for two months, at least for decisions about hiring, and while that is promising there are additional considerations with respect to employment for adults with ASD. First, many new employees engage in diversity and policy training during the first few weeks of onboarding in a new position, and that training may not be repeated during the course of an employee’s tenure with an organization. For many employees, then, training may occur many months or even years before they make a hiring decision about a given candidate with ASD. Further research is needed to understand whether the benefits of training endure over very long periods of time, and if not, to determine the ideal timeframe and process for refresher training. Second, we note that the hiring process is just the first step in the employment journey. Adults with ASD may have difficulty not only obtaining but also maintaining employment (Solomon, 2020). An educated workforce with an understanding of the complexities of ASD may be important in addressing this issue, and so neurodiversity training may be helpful not only for those involved in hiring but also for supervisors and co-workers. Further research is needed to understand the impact of such training in workforce settings and on sustained employment for workers with ASD.

## Conclusion

Employment is an essential gateway to financial stability, health care, housing, personal identity, and purpose. Research demonstrates that jobs are also an important source of social connection and can improve perceptions of well-being (Helliwell et al., 2018; Olivet Nazarene University, 2019). Individuals with ASD have significantly higher rates of unemployment and underemployment relative to both NT adults and even individuals with disabilities (Howlin, 2013; Neary et al., 2015; Roux et al., 2013, 2015; Taylor et al., 2015). One barrier to employment is the job interview, as candidates with ASD may exhibit marked differences in their social style and ability to connect with others, making them less likely to be selected for employment (Flower et al., 2021; Hedley et al., 2018; Norris et al., 2024; Whelpley & May, 2023). Recent research suggests that one approach to leveling the playing field for candidates with ASD is to couple enhanced information about ASD with diagnostic disclosure (Flower et al., 2021; May et al., 2025). The present study sought to extend these preliminary findings to determine whether positive hiring outcomes are observed with extended time between exposure to additional knowledge about ASD and the job interview evaluations. We recruited both Prolific participants from the general population and college students and asked them to evaluate job candidates with and without ASD either two weeks or two months, respectively, after completing neurodiversity training. Our findings show that although candidates with ASD continued to be rated unfavorably on some social measures relative to NT candidates, they were rated as highly as NT candidates on others, and most significantly they were equally likely to be hired as NT candidates. This pattern was observed at both the two-week and the two-month delay, suggesting that providing neurodiversity training in advance and disclosing diagnostic information at the time of interview, may be an effective strategy for supporting positive hiring outcomes for employees with ASD.
